# Provider Perceptions of Video Telehealth in Home-Based Primary Care During COVID-19

**DOI:** 10.1093/geroni/igab046.2054

**Published:** 2021-12-17

**Authors:** Emily Franzosa, Abraham Brody, Bruce Leff, Christine Ritchie, Bruce Kinosian, Alex Federman, Katherine Ornstein, Ksenia Gorbenko

**Affiliations:** 1 Icahn School of Medicine at Mount Sinai, Icahn School of Medicine at Mount Sinai, New York, United States; 2 NYU Hartford Institute for Geriatric Nursing, New York, New York, United States; 3 The Center For Transformative Geriatric Research, Johns Hopkins School of Medicine, Baltimore, Maryland, United States; 4 Massachusetts General Hospital, Boston, Massachusetts, United States; 5 University of Pennsylvania Perelman School of Medicine, Philadelphia, Pennsylvania, United States; 6 Icahn School of Medicine at Mount Sinai, New York, New York, United States

## Abstract

The COVID-19 pandemic accelerated the adoption of virtual care. In this qualitative study, we sought to determine provider perceptions of video telehealth during the first wave of COVID-19 in NYC to inform practice for home-based primary care providers nationwide. We conducted semi-structured interviews with clinical directors, program managers, nurse practitioners, nurse managers, and social workers at 6 NYC practices (N=13) in spring 2020. We used combined open and focused coding to identify themes. Participants employed both hospital-supported and commercial technological platforms to maintain care during COVID-19. Benefits of video telehealth included improved efficiency, capacity and collaboration between providers. Barriers included patients’ physical, cognitive or technological abilities, dependence on caregivers and aides to facilitate video visits, challenges establishing trust with new patients and addressing sensitive topics over video, and concerns over missing important patient information. Considering patient, clinical, and technological conditions can help optimize telehealth implementation among older homebound adults.

